# The horizontal ladder test (HLT) protocol: a novel, optimized, and reliable means of assessing motor coordination in *Sus scrofa domesticus*

**DOI:** 10.3389/fnbeh.2024.1357363

**Published:** 2024-03-06

**Authors:** Xiaobo Liu, Ana G. Gutierrez, Arlette Vega, Joshua O. Willms, Jackson Driskill, Praneetha Panthagani, Jordan Sanchez, Monica Aguilera, Brittany Backus, Jeremy D. Bailoo, Susan E. Bergeson

**Affiliations:** ^1^Department of Pharmacology and Neuroscience, School of Medicine, Texas Tech University Health Sciences Center, Lubbock, TX, United States; ^2^Department of Cell Biology and Biochemistry, School of Medicine, Texas Tech University Health Sciences Center, Lubbock, TX, United States; ^3^Department of Animal and Food Science, Texas Tech University, Lubbock, TX, United States

**Keywords:** horizontal ladder test, HLT, miniature pigs, ethanol, alcohol use, *Sus scrofa domesticus* (swine), motor coordination, validation

## Abstract

Pigs can be an important model for preclinical biological research, including neurological diseases such as Alcohol Use Disorder. Such research often involves longitudinal assessment of changes in motor coordination as the disease or disorder progresses. Current motor coordination tests in pigs are derived from behavioral assessments in rodents and lack critical aspects of face and construct validity. While such tests may permit for the comparison of experimental results to rodents, a lack of validation studies of such tests in the pig itself may preclude the drawing of meaningful conclusions. To address this knowledge gap, an apparatus modeled after a horizontally placed ladder and where the height of the rungs could be adjusted was developed. The protocol that was employed within the apparatus mimicked the walk and turn test of the human standardized field sobriety test. Here, five Sinclair miniature pigs were trained to cross the horizontally placed ladder, starting at a rung height of six inches and decreasing to three inches in one-inch increments. It was demonstrated that pigs can reliably learn to cross the ladder, with few errors, under baseline/unimpaired conditions. These animals were then involved in a voluntary consumption of ethanol study where animals were longitudinally evaluated for motor coordination changes at baseline, 2.5, 5, 7.5, and 10% ethanol concentrations subsequently to consuming ethanol. Consistent with our predictions, relative to baseline performance, motor incoordination increased as voluntary consumption of escalating concentrations of ethanol increased. Together these data highlight that the horizontal ladder test (HLT) test protocol is a novel, optimized and reliable test for evaluating motor coordination as well as changes in motor coordination in pigs.

## 1 Introduction

While rodents are the most prevalently used animal in preclinical animal research, high rates of translational failure concerning drug development have brought into sharp focus the need to study mammalian species that are physiologically more similar to humans, particularly in relation to the aspects of the diseases being modeled (Public Law 89-544, 1966; Public Law 91-579, 1970; Public Law 94-279, 1976; Ioannidis, 2005, 2006; Goodman and Greenland, 2007; Chavalarias and Ioannidis, 2010; Paul et al., 2010; Bailoo et al., 2014b; Gaire et al., 2021). Pigs are an important model in preclinical biomedical research, historically accounting for approximately 6% of all the United States Department of Agriculture (USDA) species protected under the Animal Welfare Act (Public Law 89-544, 1966; Public Law 91-579, 1970; Public Law 94-279, 1976). Pigs share numerous physiological and neuroanatomical similarities with humans (e.g., Lee et al., 2013; Schomberg et al., 2017) and, consequentially, are used in the research setting for the study of oncology, cardiovascular diseases, pediatric disorders, regenerative medicine, transplantation, medical imaging, genomic and reproductive biotechnology, neurological diseases, surgical innovation, gene therapy/immunotherapy, infectious diseases, effects of microbiota, metabolism, nutrition, and education (Vodicka et al., 2005; Lunney et al., 2021).

There is a relative dearth of reliable, validated means of behavioral assessments for pigs within a biomedical setting (Gieling et al., 2011; Murphy et al., 2014). One of the commonly evaluated behavioral phenotypes in the laboratory pig is impaired motor coordination. The apparatuses used to evaluate motor coordination differences in pigs include the open field, the balance/inclined beam, and the treadmill. It has been argued that analysis of pig behavior within these apparatuses permits researchers to evaluate motor behavior within a controlled environment. However, these behavioral assessments are derived from rodent models with little emphasis on the degree to which rodents and pigs differ and the consequences of such differences on the validity of derived experimental results (Carter et al., 2001; Vodicka et al., 2005; Murphy et al., 2014; Shultz et al., 2020). For example, the inclined beam test, which is very similar to the balance beam test for rodents, can also be potentially dangerous to a pig with impaired motor or cognitive function if it were to fall. Moreover, at baseline/unimpaired levels, some animals cannot walk at least halfway up the beam, calling into question the reliability and validity of such assessments (Sullivan et al., 2013). Similarly, in the open-field test, the ability to measure locomotor behavior while inducing conflict in the emotional/arousal domains of behavior often yields conflicting results across replicate studies (Gieling et al., 2011; Murphy et al., 2014). Additionally, the typical open field arena for pigs is orders of magnitude smaller than the typical foraging distance observed within an ethological setting, calling into question the ethological validity of this test (Murphy et al., 2014). Assessment of gait using a treadmill often requires advanced equipment and facilities, which are generally cost-prohibitive and for which infrastructure is sorely lacking (Boakye et al., 2020; Shin et al., 2021). Therefore, inexpensive apparatuses that are safe to use, and protocols which are easy-to-train, and which produce reliable and repeatable evaluations of motor behavior are critically needed for assessing motor impairment in pigs.

To bridge this knowledge gap, an apparatus modeled after a horizontally placed ladder was designed. While this apparatus design may appear to be similar to the “elevated ladder beam” test employed with rats (Soblosky et al., 1997) and mice (Cummings et al., 2007), the design of the Horizontal Ladder Test (HLT) used here drew inspiration from the human standardized field sobriety test (FST), specifically the ‘walk and turn’ test (WAT) utilized for detecting driving under the influence (DUI) of alcohol (Cole and Nowaczyk, 1994; Burns, 2003). In the DUI detection test, individuals are instructed to take nine heel-to-toe steps along a straight line, followed by turning on one foot and returning in the opposite direction (Downey et al., 2016). Given the unique physical characteristics of pigs—short legs, substantial weight, and an ungainly gait (Vodicka et al., 2005; Gieling et al., 2011; Lunney et al., 2021)—the apparatus and protocol were customized to minimize physical contact with the apparatus during crossing; the operational definition of motor incoordination used here related to the degree of contact with the apparatus (see Behavioral Ethogram, Table 1). The modular design of the apparatus allowed for easy adjustment of rung height from 3 to 6 inches in 1-inch increments. The straight, linear ladder with a restricted width parallel to the ground simulated the straight line in the WAT, considering the physical and behavioral differences of pigs. The concept of “walk and turn” was incorporated into the strategy for pigs to perform double-crossings. The number and height of the rungs presented varying difficulty levels, limiting the steps the pigs could take. This approach allowed the pigs to learn to “pick up their feet” and permitted for adjustment of difficulty for testing varying hypothesis while avoiding ceiling and floor effects in terms of the number of errors made. Furthermore, the ethogram that was developed based on the eight indicators of impairment used from the walk and turn test (c.f., Table 1). These indicators included the inability to maintain balance during instructions, starting too soon, stopping while walking, failure to touch heel-to-toe, stepping off the line, using arms for balance, executing an improper turn, or taking an incorrect number of steps. It was hypothesized that: (1) by increasing the rung height to 6 inches during the initial training phase, the pigs would be forced to lift their feet in order to be able to cross the ladder to learn the task, and (2) the pigs would make significantly fewer errors as the rung height was lowered from 6 to 3 inches, in 1-inch increments across training sessions.

**Table 1:** Ethogram for the scoring of motor coordination in the HLT.

Event	Operational definitions and decision rules	Score/instance
Kick	The animal’s front or back of the leg contacts the rung of the ladder	1-point
	***Decision Rules:***	
	Kicking of the rung is often accompanied by an auditory cue that is distinguishable from other room sounds. When the leg contacts the rung, a distinct reverberation is heard given the hollow structure of the PVC tube	
	Multiple kicks within a single bar are counted separately, and if the pig fails to lift its leg sufficiently for the ladder and attempts numerous times, each kick per leg is counted	
	Crossings where the pig enters the course off-center and kicks or steps on the bar due to this off-center entry are not scored	
	If the pig’s snout touches or pushes the PVC tube, it is also considered a kick, but it must be accompanied by a corresponding movement of the bucket in the direction that the pig crosses	
Step	The animals’ heel or sole of the hind hoof (plantar surface) contacts the rung of the ladder	2-points
	***Decision Rules:***	
	The behavior looks similar to kicking, but is specific to the orientation of the leg upon contact with the apparatus	
	An auditory cue, similar to “Kick” may be heard. The distinguishing criteria relates to the animal momentarily pausing, with the pause coinciding with a postural drop of the animal’s back due to the mis-step	
	Multiple steps on a single tube are counted separately	
	To measure a step, the pig’s foot must be positioned directly above the tube and remain there for at least 1 s. If the foot slides off the tube, it is considered a kick, not a step	
Body Hit	The animal’s body, excluding the leg, contacts the apparatus, resulting from a slip (see event below)	3-points
	***Decision Rules:***	
	In cases where a slip leads to a body hit, both the slip and the body hit are scored	
	Multiple body hits on the same bucket are counted as separate events	
	Signs of an unsteady gait, such as wobbling, stumbling, slipping, or tripping, must be observed in conjunction with a body hit	
	Events where the pig bumps into the bucket before starting the crossing (head or shoulder) or at the end (rump) due to turning are not considered body hits	
Slip	A slip is characterized by a momentary loss of balance, such that the animal is able to continue crossing the ladder in a given pass	4-points
	***Decision Rules:***	
	The hind leg(s) may slide on the floor, without the animal falling to the ground, but there is an observable drop in the pig’s back	
	A body hit on a bucket may occur simultaneously, in which case both the slip and the body hit are counted	
Fall	A fall event occurs when the pig loses balance to the extent that a body part, other than the legs, contacts or touches the ground. During a fall, the pig comes to a complete stop and may struggle to regain its footing	5-points
	***Decision Rules:***	
	If a pig falls at the first bar and cannot complete the course, there is an automatic deduction of 20 points, equivalent to four falls	
	If the pig falls closer to the middle of the course and cannot continue, an additional fall is recorded for each tube ahead	

As an initial validation of construct validity, changes in motor coordination as a consequence of voluntary consumption of escalating concentrations of alcohol (ethanol) in water was evaluated. It was predicted that, similarly to humans, as the percentage of ethanol in water consumed increased from 2.5 to 10% during voluntary drinking sessions, a significantly greater number of motor coordination errors would be observed when performing the HLT task. By conducting training and testing on flat ground, the risk of injury to the pigs, even when they were heavily intoxicated, was minimized. The HLT protocol can therefore be used to evaluate motor coordination across a wide array of disease models in pigs. This includes applications in the pre-and post-treatment evaluation for substance use disorders including AUD, neurodegenerative diseases such as amyotrophic lateral sclerosis (ALS) and Parkinson’s disease (PD), motor impairments resulting from conditions including multiple sclerosis (MS), strokes, traumatic head injuries, and ataxic disorders of balance, vestibular function and proprioception.

## 2 Materials and equipment

All materials and equipment used in this protocol are listed in Table 2.

**Table 2:** List of materials and equipment used to conduct the HLT.

Name of material/equipment	Company	Catalog number	Comments/descriptions
5 Gallon Orange Homer Bucket	The Home Depot	05GLHD2	3-pack for $12.10. 13 buckets in total, 8 for ladders, 5 for blocking
1–1/4′′ Schedule 40 White PVC Pipe 4,004-012AB - 5 ft	JM EAGLE	4004-012AB	$10.11 per unit. Two 5 ft. PVC tubes in total needed for the ladder
40 lbs. Premium Plaster Sand - Filtered, Screened and Washed Fine Sand Common Ingredient in Mixing Mortars	The Home Depot	02-0509	$80.99 per 40 lbs. Twelve bags needed in total. This can be substituted by other kind of weights that are cheaper, for example, dumbbells, bowling balls, rocks/stones/dirt/sand from outside, etc.
Samsung F90 White Camcorder with 2.7′′ LCD Screen and HD Video Recording	Samsung	N/A	Two cameras needed. From $68 to $100 on eBay. Is discontinued by manufacturer
SanDisk 64GB Extreme SDXC UHS-I Memory Card - C10, U3, V30, 4 K, UHD, SD Card - SDSDXV2-064G-GNCIN	Amazon	B09X7DY7Q4	$12.42/ea., two SD cards needed
Aracombie 4PCS 1–1/4 Inch to 2-1/8 Inch Hole Saw Kit for Wood	Amazon	B09SSX9QYS	$8.99/ea. 1.2 Inch Cutting Depth Hole Cutter Drill Bit with Mandrel, Round Hole Saw Set for Cornhole Boards Plastic Drywall Soft Metal (Gray)
BLACK+DECKER 20 V MAX* POWERECONNECT Cordless Drill/Driver +30 pc. Kit (LD120VA)	Amazon	B006V6YAPI	$59.99/ea.

## 3 Protocol

### 3.1 Animal husbandry

Five female Sinclair miniature pigs purchased from Sinclair BioResources (Auxvasse, MO) at 7 months of age were individually housed and acclimated to the local laboratory environment. Before entering the experiment, the pigs were allowed to reach maturity, approximately 1.5 years of age which is equivalent to approximately 21 human years (Age Converter, 2015). The training procedure took 2 weeks and testing occurred over 2 months. In the validation study, testing occurred 1 year subsequent to training due to delays associated with the COVID-19 pandemic. Animals were housed in an environmentally controlled vivarium with an 11/13 light/dark cycle, with lights off at 18:00. The temperature in the housing room was maintained at 23°C–25°C, with humidity levels set to 30–60%. The Laboratory Animal Resources Center staff provided the pigs with two meals daily (LabDiet^®^ 5081, Richmond, IN), at 08:00 and 13:30, during weekdays, while a single meal of the same type was given at 08:00 on weekends, with twice the usual weekday food quantity allotment. Access to filtered tap water was unrestricted for the pigs, except during specific two-bucket, free-choice alcohol access testing periods when house water was temporarily turned off (see Section 3 below). All experimental procedures conducted in this study were approved by the Institutional Animal Care and Use Committee at Texas Tech University Health Sciences Center. Our institution, and thus our approved protocol, maintains compliance with federal guidelines (the Public Health Service, the Office of Laboratory Animal Welfare and the USDA) as well as via voluntary accreditation with the Association for Assessment and Accreditation of Laboratory Animal Care (AAALAC).

### 3.2 The horizontal ladder test—description and protocol

#### 3.2.1 Apparatus description

**Figure 1:**
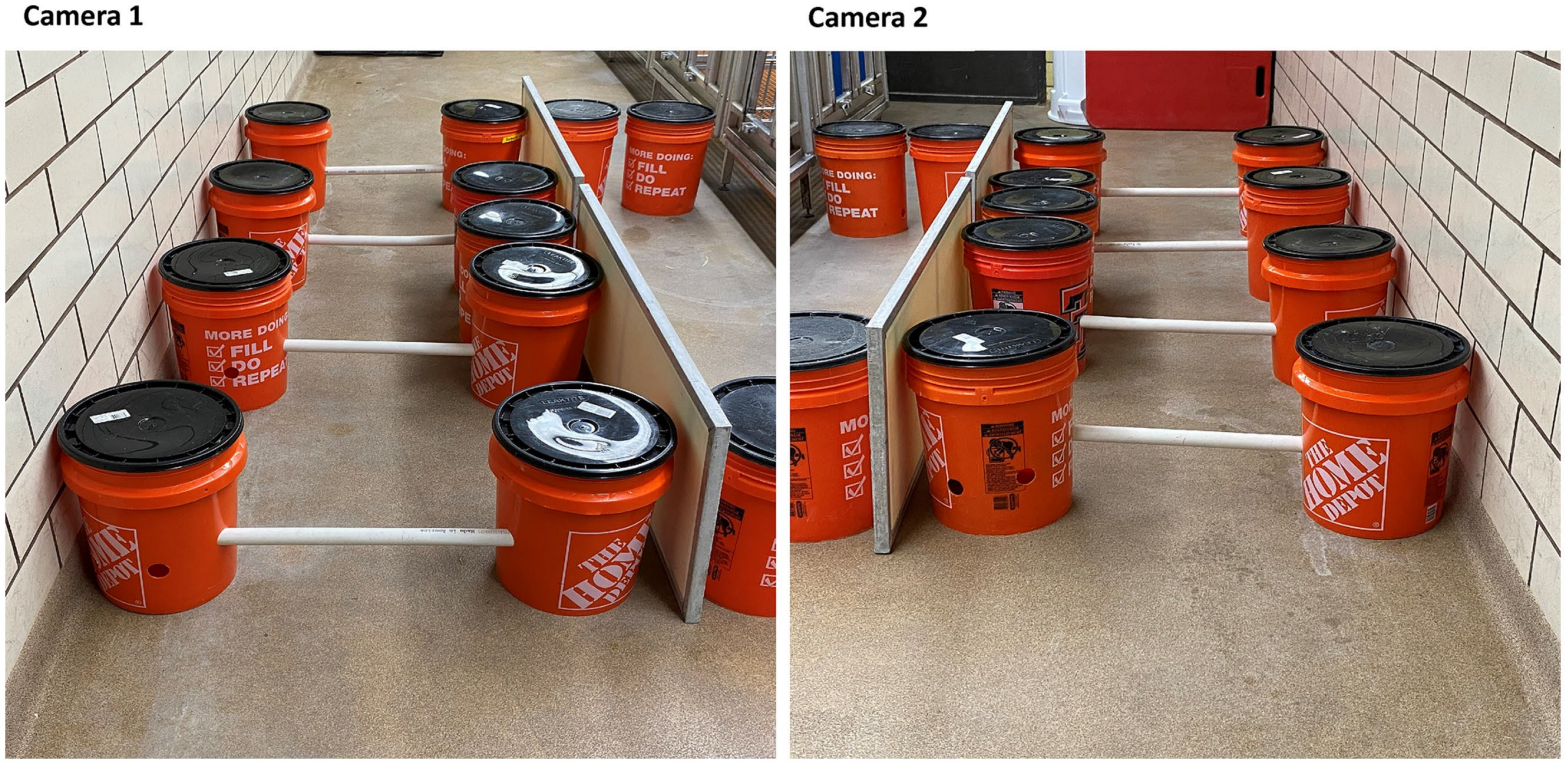
The horizontal ladder setup with exemplars of the achieved camera-view from both sides.

**Figure 2:**
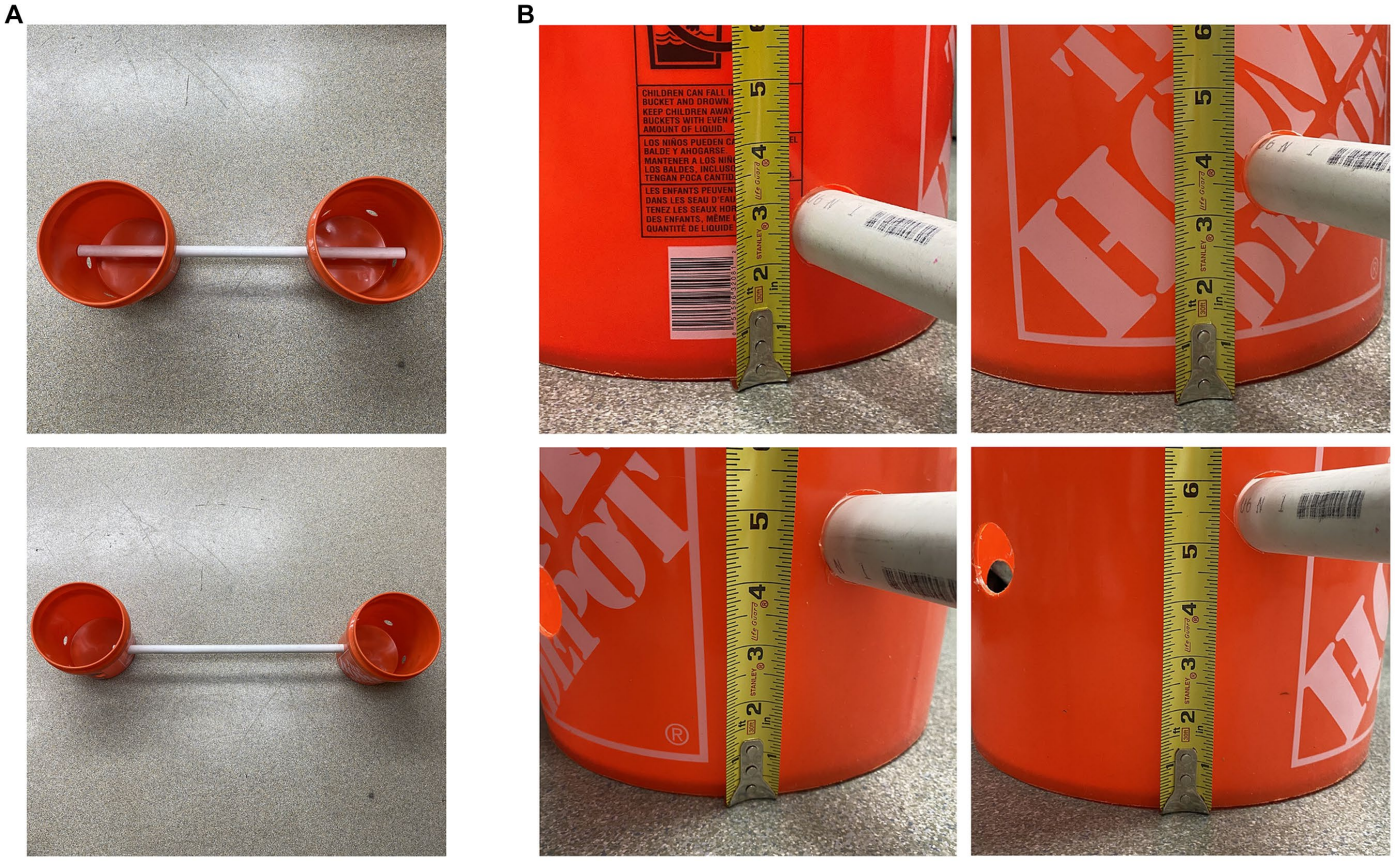
Example images showing the adjustable length and height of the ladder rungs. **(A)** The upper picture shows the shortest length of the rung, and the lower picture shows the longest length of the rung. **(B)** From the top left to the bottom right, the rung heights were at 3 in, 4 in, 5 in, and 6 inches with holes drilled with spaces around each bucket.

The horizontal ladder apparatus is comprised of four rungs and eight 5-gallon buckets (Figure 1). The rungs of the horizontal ladder apparatus are comprised of lengths of Schedule 40 polyvinyl chloride (PVC) tubes, 1.5 inches in diameter and 88.5 inches in length (to accommodate the width of the largest pig used in our study). The length of PVC tubes used can be increased or shortened depending on the size of the animal in a given study (Figure 2A). Each rung is capable of being slotted into 5-gallon buckets at a height of 3, 4, 5, or 6 inches (Figure 2B) from the floor through 1.5-inch diameter drilled holes. At most, the fall distance for a pig is 6 inches, minimizing, if not essentially eliminating, the potential for injury. Each bucket is weighted with 35 lbs. sandbags for stability and to minimize the deviation of our pre-specified rung distance interval of 29.5 inches (the maximum length of our pigs). Both the weight of the buckets and the inter-bucket distance can be adjusted as needed (see Section 3.2.2 below). The total cost of the apparatus to build, using materials readily available at a big-box hardware store, was less than $250.00 excluding the sand, which can be substituted for other commonly available weight sources (e.g., rocks; see Table 2 for details).

#### 3.2.2 Apparatus setup

1. Place the rungs of the ladder into each 5-gallon weighted bucket at either 6, 5, 4, or 3 inches, depending on the training day. Attach the other end of the PVC tube to another bucket so that each end of the rung is completely within the bucket.
2. Arrange the assembled buckets in a parallel fashion on the floor, with a gap of 29.5 inches—or the width of your largest pig—between them. Laboratory tape may be used on the floor to designate this distance and for easy replacement of the buckets should they be moved. The width of the tubes can be easily adjusted by sliding the tube more or less into the bucket (±10 inches in each bucket, 20 inches total) to accommodate the average width of each pig. The sides of the ladder can be blocked using easily obtainable materials, such as additional weighted buckets, or by placing one side of the ladder against a wall. The accommodations effectively ensure that the animal is only able to traverse the ladder by proceeding forward or backward across the ladder, with no opportunity for “exiting” the ladder until the last rung is reached.
3. Set up the tripods and video cameras, one on each end of the ladder, such that the view of the animal is unobstructed. Ideally, the cameras should record video in a minimum resolution of 720p and with stereo audio. Refer to Figure 1 for representative images of the assembled ladder and setup of the camera viewpoints.
4. Position one investigator at the far end of the ladder while a second investigator stands at the opposite end of the ladder. Each investigator can encourage the animal to cross the ladder using food rewards (e.g., marshmallows, breakfast cereals, etc.). The same food reward and quantity should be used throughout the experiment so that any variation with anticipation of reward is equally distributed across all experimental subjects.

#### 3.2.3 Behavioral training

**Figure 3:**
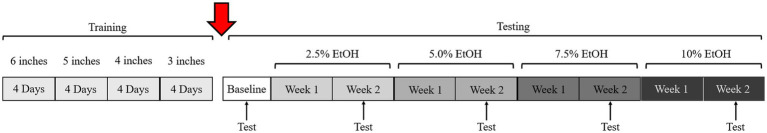
Timeline of training and testing. The transition from training to testing can vary. In this study, testing was delayed by 1 year due to the ongoing COVID-19 pandemic (red arrow). Similar baseline data during training and 1-year-later during testing/validation suggest that animals remembered the task well (c.f., Figures 4, 6).

Training to cross the ladder proceeds in a stepwise process, which minimizes the number of things that the animal has to learn in a single step, and which also minimizes the potential for off-task behavior. Such a stepwise process also permits re-training at a specific step if recalcitrant behavior is observed. In the present study, each step required 4 days for training to be completed (see Figure 3). A single medium-sized marshmallow was given after each double-crossing as a reward.

##### 3.2.3.1 Step 1

- An investigator guides each pig to cross the ladder, offering treats as a reward whenever a pig completes a single pass of the ladder. During this first step, the rungs are initially set to the highest level of 6 inches, so that the animal is forced to lift its legs in order to cross.
- Animals are only rewarded when they complete two passes of the ladder, with the rung height set to 6 inches, before returning to their initial starting location. The second investigator assists in turning the animal around once the animal has completed its first pass. This step continues until the animal can, at a minimum, make 3 continuous double crossings (out and back) of the ladder.

##### 3.2.3.2 Step 2

- Set the rung height to 6 inches and have the animal perform three double crossings at this rung height, before reducing the rung height to 5 inches. This step is to reinforce the learned behavior of the previous step while making the difference in rung height more salient when it is lowered.
- Animals are only rewarded when they complete a double crossing of the ladder (out and back once), with the rung height set to 5 inches, before returning to their initial starting location. The second investigator assists in turning the animal around once the animal has completed its first pass. This step continues until the animal can, at a minimum, make 3 continuous double crossings of the ladder. If off-task behavior is observed (e.g., refusal to cross), re-train the animal on Step 1.

##### 3.2.3.3 Step 3

- Set the rung height to 5 inches and have the animal perform three double crossings at this rung height, before reducing the rung height to 4 inches. This step is to reinforce the learned behavior of the previous step while making the difference in rung height more salient when it is lowered.
- Animals are only rewarded when they complete a single double crossing of the ladder, with the rung height set to 4 inches, before returning to their initial starting location. The second investigator assists in turning the animal around once the animal has completed its first pass. This step continues until the animal can, at a minimum, make 3 continuous double crossings of the ladder. If off-task behavior is observed (e.g., refusal to cross), re-train the animal on Step 2.

##### 3.2.3.4 Step 4

- Set the rung height to 4 inches and have the animal perform three double crossings at this rung height, before reducing the rung height to 3 inches. This step to reinforce the learned behavior of the previous step while making the difference in rung height more salient when it is lowered.
- Animals are only rewarded when they complete two passes of the ladder, with the rung height set to 3 inches, before returning to their initial starting location. The second investigator assists in turning the animal around once the animal has completed its first pass. This step continues until the animal can, at a minimum, make 3 continuous double crossings of the ladder. If off-task behavior is observed (e.g., refusal to cross), re-train the animal on Step 3.
  Training is considered complete when the pigs consistently score less than 4 points during a single crossing at a rung height of 3-inches (see Table 1 for scoring criteria).

#### 3.2.4 Video scoring

##### 3.2.4.1 Video data preparation

Videos should be screened for excessive noise, completeness (e.g., equipment failure), and other technical issues (e.g., improper placement of cameras). Only complete data for each pig should be analyzed. In cases where animals belong to experimental groups (e.g., treated vs. control), videos can be renamed so that the experimenter is blinded to treatment. It may not always be possible to blind the experimenter to treatment if, for example, the hair/bristle of the pigs are different colors. Where possible, the experimenters who test the animals should be different from those who code the video data to avoid bias.

##### 3.2.4.2 Behavioral ethogram

An ethogram, which included operational definitions, decision rules and scoring criteria, was developed for the scoring motor coordination behavior that can be generalized to other laboratory/vivarium settings (see Table 1). The ethogram focused on five observable and objective criteria: kicking (of the rungs or buckets), stepping (on the rungs), body contact with the apparatus (rungs or buckets), slipping (momentary loss of balance), and fall (complete loss of balance with contact of the body unto the floor) (c.f., Table 1, Supplementary videos S1–S11). The degree of motor incoordination was represented in each of the criteria with, for example, the kicking of a rung being less severe (1-point) than falling (5-points). Data were scored using the open-source software Behavioral Observation Research Interactive Software (BORIS), and previously established methods for reliability assessment (Bailoo et al., 2014a, 2018a,b, 2020; Varholick et al., 2018, 2019). For these experiments, inter-and intra-rater reliability was established at 80% concordance (Jansen et al., 2003). The primary outcome measure from these evaluations was the total score, at each rung height, across three double crossings. Here, higher scores reflect a greater number of motor coordination errors.

### 3.3 Evaluation of construct validity using voluntary ethanol consumption

#### 3.3.1 The two-bucket choice test

The pigs were given a choice between two buckets: one containing water and the other with increasing alcohol (ethanol, or EtOH) concentrations, starting at 2.5% and progressing to 5%, then 7.5%, over 2 weeks for each concentration. Subsequently, the ethanol concentration was elevated to 10%, remaining at this level for 8 weeks. The location of water and alcohol buckets (left or right side) was alternated daily to control for side-biased responding. Animals had access to alcohol from 10:00 to 17:00 each day, with a maximum volume limit of 3.5 liters, aiming to prevent discontinuous alcohol consumption or any potential life-threatening situations arising from the adverse effects of excessive alcohol intake. After 17:00, bucket access was stopped, and house water was reinstated until the next round of alcohol exposure.

#### 3.3.2 Evaluation of motor coordination errors

1. Before collecting baseline data, animals were trained to cross the ladder using the methods described in Section 2.
2. Baseline data, at a rung height of 3 and 6 inches, was then collected 1 week prior to the commencement of alcohol exposure.
3. The animals were then evaluated for motor coordination errors in the HLT at a rung height of 3 and 6 inches for each alcohol concentration, and the data was compared to baseline levels (refer to Figure 3 for timeline of data collection).

#### 3.3.3 Blood alcohol concentration determination

1. Blood samples were obtained from the pigs’ ear veins approximately 1 h after they had concluded their daily drinking sessions for each alcohol concentration exposure. The pigs were securely restrained to minimize head movement, and blood draws were conducted using a 1 mL syringe with a 25Ga, ½ inch needle through an ear vein. Blood sampling occurred once during the second week of exposure at each alcohol concentration for a total of 4 samples in 2 months.
2. Here, 50 μL of blood was placed into a Gas Chromatography (GC) vial along with 100 μL of water, and the vial cap was securely sealed to prevent any vapor leakage.
3. Prior to GC analysis, these vials were stored on ice.
4. For quality analysis and quality control (QAQC) evaluation, a series of standard solutions (0, 0.025, 0.5, 1, and 2 mg/mL) were analyzed along with the actual samples.
5. The BACs were then analyzed using GC (Syapin et al., 2016).
6. The final BAC values were computed based on the resulting standard curve.

### 3.4 Statistical analyses

All statistical analyses were conducted in GraphPad Prism v10.0.3. Depending on the sample size and adherence to distributional assumptions, a within-subject ANOVA may be used for longitudinal analysis of changes in motor coordination behavior. In this study, a non-parametric Friedman test for analysis of repeated measures data was used, followed by *post hoc* corrected Dunn’s test to evaluate motor coordination deficits in the HLT. For analysis of BAC, the median of each group was compared with the standard value for intoxication (0.8 mg/mL) using a one-sample *t*-test. Simple linear regression was used to evaluate the relationship between average motor coordination errors and average alcohol consumption.

## 4 Representative results

**Figure 4:**
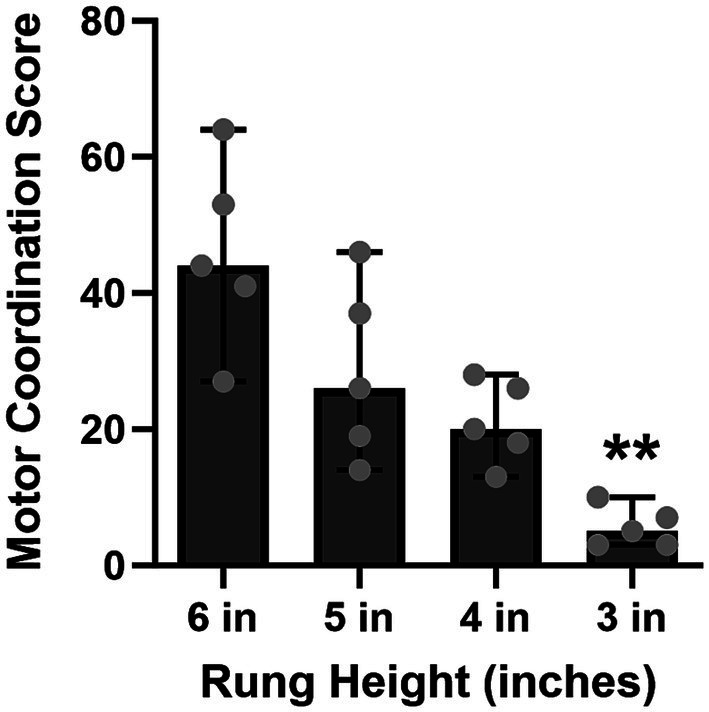
As the rung height was decreased across training, the motor coordination score significantly decreased (6 in vs. 3 in, *p* = 0.0014) and was less variable—that is, the animals performed significantly better with fewer errors at the 3-inch rung height. Bars represent medians ±95% CI. **p < 0.01.

**As predicted, the motor coordination score in the HLT decreased as the rung height was decreased from 6 to 3 inches** [χ^2^(4) = 14.04, *p* = 0.0014, Figure 4].

A sequential training approach was implemented as detailed in Section 2 of the HLT protocol, where the rung height of the ladder gradually decreased from 6 inches to 3 inches. Figure 4 illustrates results of the final day of training at each specific rung height. Initially, the motor coordination score in the HLT was higher (more errors) and more variable at 6 and 5 inches. However, as training progressed, each pig’s performance became more consistent, with lower variability and a lower score (fewer errors), at the 3-inch rung height. On the last training day at the 3-inch height, the median score was low, at 5 points from the total of 3 double crossings. These data suggested that the specified training strategy was effective—the pigs completely lifted their legs when crossing which, in turn, minimized the number of motor coordination errors.

**Figure 5:**
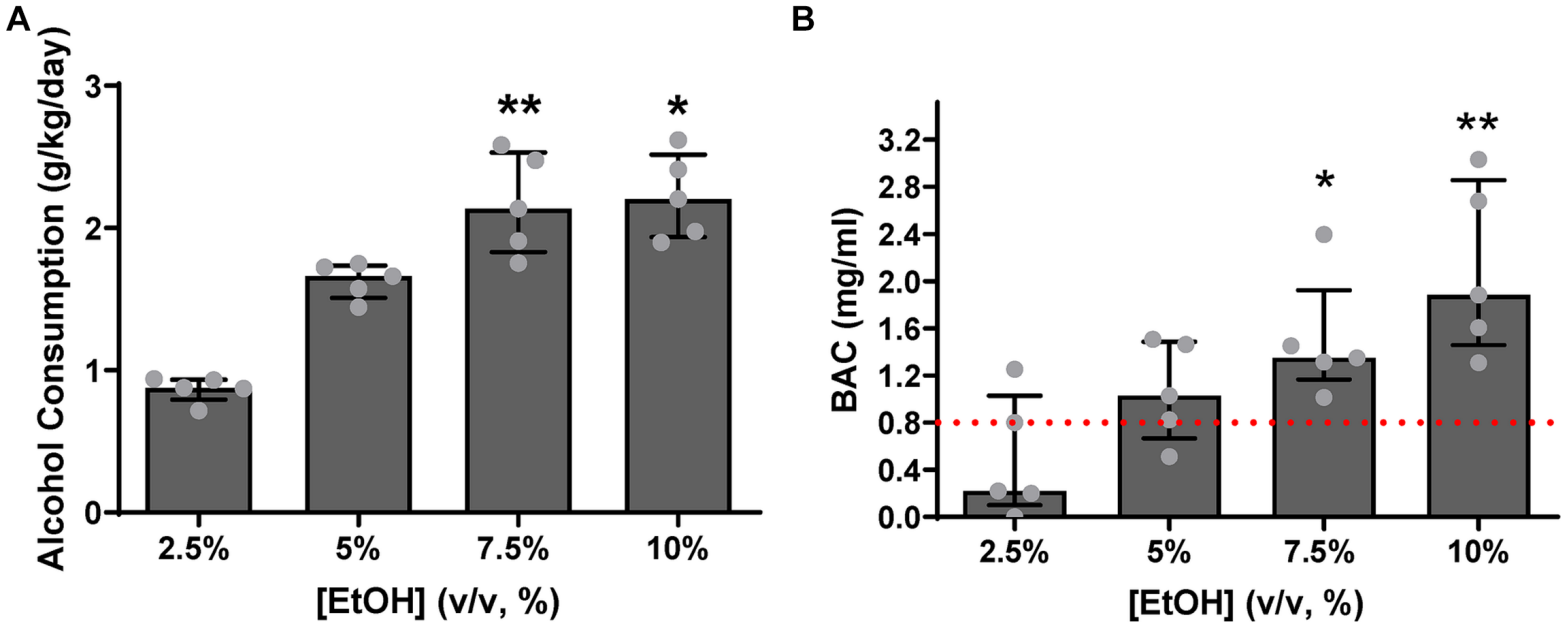
**(A)** Animals consumed more alcohol, with increasing alcohol concentration, significantly escalating their intake at 7.5% (*p* = 0.0087) and 10% (*p* = 0.0197) relative to 2.5% levels. **(B)** At 2.5% concentration levels, two pigs reached the intoxication level criterion defined by NIAAA, while at 5%, four pigs achieved criterion. All pigs reached intoxication criterion at 7.5 and 10%, with a statistically significant difference being observed compared to the standard value of intoxication (0.8 mg/mL) at 7.5 and 10% ethanol concentration (*p* = 0.0396 and *p* = 0.0162, respectively). [EtOH] represents alcohol concentration, % volume to volume. Bars represent medians ±95% CI. *p < 0.05; **p < 0.01.

**The pigs drank a larger amount of alcohol as alcohol concentration increased** [χ^2^(4) = 13.56, *p* = 0.0001, Figure 5A] **and drank to intoxication levels** (BAC > 0.8 mg/mL).

As shown in Figure 5A, a significant increase in alcohol consumption was observed at alcohol concentrations of 7.5% [*Z* = 3.184, *p* = 0.0087] and 10% [*Z* = 2.939, *p* = 0.0197], relative to 2.5% concentration levels. Two pigs drank to intoxicating levels, 0.8 mg/mL (0.08 g/dL), as defined by National Institute on Alcohol Abuse and Alcoholism (NIAAA) (NIAAA, 2023), already at 2.5% alcohol concentration levels. As shown in Figure 5B, as alcohol concentration increased, four pigs reached the intoxication criterion at 5% alcohol concentration levels, and all pigs reached criterion at 7.5 and 10% alcohol concentration levels [*t*(4) = 3.008, *p* = 0.0396 and *t*(4) = 3.993, *p* = 0.0162, respectively]. Thus, motor coordination deficits as a consequence of consuming alcohol to intoxicating levels was evaluated next.

**The number of motor coordination errors increased with increased alcohol consumption**.

**Figure 6:**
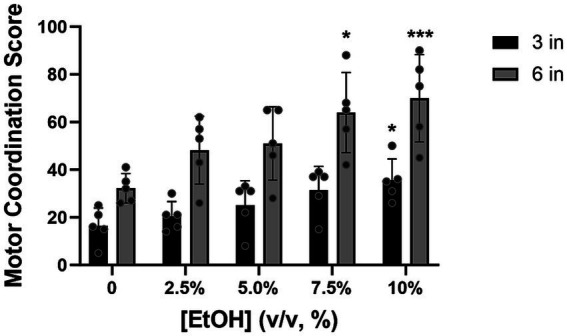
At 3 inches, the motor coordination score increased as alcohol concentration increased, with a statistically significant difference, relative to baseline, being observed at concentrations of 10% (*p* = 0.0201). At 6 inches, the test score showed a similar pattern of increasing and showed significance at 7.5% (*p* = 0.0277) and 10% alcohol concentrations (*p* = 0.0004) compared to baseline (2.5%). [EtOH] represents alcohol concentration, % volume to volume. Bars represent medians ±95% CI. *p < 0.05; **p < 0.01.

All the animals showed good recollection of the HLT task, 1 year later, with the median score being lowest at baseline (i.e., no alcohol exposure). During testing, as the concentration of alcohol increased, the score in the HLT also increased (Figure 6). Consistent with our BAC data, the highest motor coordination scores at 3 inches were observed at 7.5 and 10% alcohol concentrations, with a statistically significant difference being observed between baseline and the 10% alcohol concentration [*Z* = 2.8, *p* = 0.0204]. A similar pattern of performance was also observed at 6 inches (the highest rung height), with a statistically significant difference relative to baseline being observed at 7.5% [*Z* = 2.7, *p* = 0.0277] and 10% [*Z* = 3.9, *p* = 0.0004] alcohol concentrations. The median baseline score, already high at 6 inches, increased further with the escalation of alcohol, suggesting that excessive alcohol consumption exacerbated the already challenging task.

**Figure 7:**
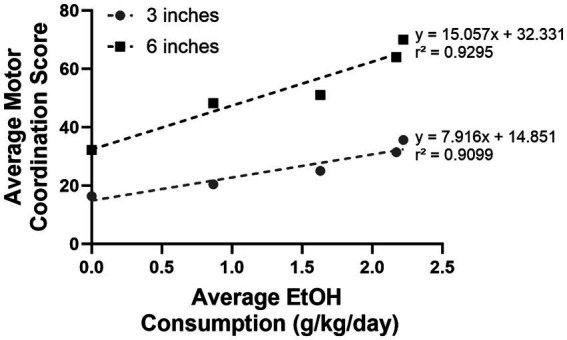
A significant linear relationship was observed between the average motor coordination score and average alcohol consumption at both 3 and 6 inches; alcohol consumption continuously increased as alcohol concentration increased. Each data point in the graph above represents the average alcohol consumption and the corresponding average motor coordination score at 0, 2.5, 5, 7.5, and 10% EtOH, respectively.

A positive linear relationship between motor coordination and escalating alcohol consumption was observed at both the 3- and 6-inch rung height ([*F*(1,3) = 30.28, *p* = 0.0118 and *F*(1.3) = 39.55, *p* = 0.0081], respectively; Figure 7); as alcohol concentration increased, the motor coordination score also increased. The proportion of variance (r^2^) accounted by the fit model was 91 and 93% at 3 and 6 inches, respectively. Notably, a steeper slope was observed when comparing the relationship between motor coordination and alcohol concentration at 6 inches compared to the 3 inches [*β* = 15.057 vs. 7.916]. This result highlights that crossing the ladder at higher rung heights is more difficult than at lower rung heights.

A remarkably low number of errors was observed after training at the 3-inch height, and the data exhibited uniformity. Even a year post-training, the pigs consistently demonstrated minimal errors at the 3-inch height, surpassing expectations (a minimum of 4 points during a single crossing, resulting in a total score of 24 points with 3 double-crossings; the average baseline performance was 16 points). Furthermore, the pattern of task performance remained consistent across various heights (Figure 6). This suggests that proficiency at the 3-inch level is not solely due to the ease of the task at this rung height, but rather indicates a genuine understanding of the task and retention of the learned information. Additionally, under challenging circumstances where animals may be severely impaired and unable to perform at the 6-inch height, the HLT remains a reliable, easy, and safe measure, height adaptable as needed.

## 5 Discussion

The primary objective of this study was to develop and validate a bespoke HLT apparatus and protocol as a means of evaluating motor coordination deficits in pigs, given their physiological similarities to humans as compared to rodents (Vodicka et al., 2005; Gieling et al., 2011; Lee et al., 2013; Sullivan et al., 2013; Murphy et al., 2014; Schomberg et al., 2017; Lunney et al., 2021). The data, albeit with a small sample size, are consistent with our predictions. During training, the number of errors observed in the HLT decreased as the rung height decreased. This difference while potentially attributable to the test being easier to perform at 3 inches, may also be attributed to the animals understanding the requirements of the task likely due to the forced higher lifting of their legs at the 6-inches rung height. Tentative evidence supporting the latter contention, however, may be gleaned from the observation of a similar pattern of performance, under varying alcohol concentration exposures, at both 3- and 6-inches rung heights (Figure 6). Further research may assist in adjudicating between these two alternative explanations. Given the short legs and heavy body weight of the pigs, training the animals at a rung height of 6 inches, followed by a gradual decrease to 3 inches across training sessions, is sufficient to provide a reasonable baseline measurement of motor coordination.

A secondary objective of this study was to perform an initial evaluation of construct validity of the HLT protocol. Consistent with our predictions, the same animals that were successfully trained on this protocol during the primary objective evaluations had higher motor coordination scores (i.e., made more errors) as ethanol concentration and consumption to intoxication increased. These results are consistent with other studies in mice and rats which demonstrate a greater degree of motor incoordination at high levels of ethanol concentration and/or consumption (e.g., Middaugh et al., 1992; Hanchar et al., 2005; Bohlen et al., 2014). The overall pattern of motor incoordination was consistent at both 3- and 6- inches; as alcohol consumption increased the motor coordination score similarly increased (more errors/contact with the apparatus). Nevertheless, individual differences in motor incoordination were observed, likely attributable to differences in individual pigs’ drinking behavior—both in volume and speed of consumption. It is conceivable, however, that these individual differences in drinking could be eliminated if a fixed volume and concentration of alcohol were administered via gavage. However, this protocol was developed as part of a larger project, which evaluates progression to Alcohol Use Disorder (AUD) at an individual level, and thus, a decision to use a free-choice alcohol consumption paradigm was made (Liu et al., 2023). Together, these results demonstrated that our HLT protocol shows good face and construct validity, as it is both suitable for evaluation of motor coordination in pigs as well as sensitive to deficits in motor coordination as a consequence of ethanol consumption.

The decision to exclusively use female pigs in this study was also influenced by the overarching research question (Liu et al., 2023) and limitations in terms of available space at our vivarium. Given that this was a pilot study focused on method development and prioritizing investigators’ safety, female pigs were chosen for their generally more docile behavior upon reaching puberty. Correspondingly, male pigs can display agonistic behavior at puberty, particularly when housed in close vicinity to female conspecifics (Vodicka et al., 2005; Lunney et al., 2021). While our decision to study female pigs was therefore pragmatic, we do not predict that sex-differences in motor coordination would be observed in subsequent studies.

While using the pig as a model organism has translational advantages; they are costly, require significant space and infrastructure as well as trained personnel in the management and welfare of large mammals. In contrast, a significant benefit of our apparatus is that it is considerably cheaper to make and to perform than many automated rodent devices. The materials for the HLT apparatus can be obtained from any big box hardware store. In addition, it allows adjustments for size and difficulty and represents a low risk of injury even when animals were heavily intoxicated. We, therefore, confidently recommend the HLT protocol for use across a wide array of disease models in pigs and posit its adaptation and use for other large animal species.

## Data availability statement

The raw data supporting the conclusions of this article will be made available by the authors, upon reasonable request.

## Ethics statement

The animal study was approved by Institutional Animal Care and Use Committee at Texas Tech Health Sciences Center, Lubbock, TX. The study was conducted in accordance with the local legislation, federal guidelines, and institutional requirements.

## Author contributions

XL: Conceptualization, Data curation, Formal analysis, Investigation, Methodology, Project administration, Supervision, Validation, Visualization, Writing – original draft, Writing – review & editing. AG: Formal analysis, Investigation, Writing – review & editing. AV: Formal analysis, Investigation, Writing – review & editing. JW: Investigation, Writing – review & editing. JD: Investigation, Writing – review & editing. PP: Investigation, Writing – review & editing. JS: Investigation, Writing – review & editing. MA: Investigation, Writing – review & editing. BB: Conceptualization, Resources, Writing – review & editing. JB: Conceptualization, Formal analysis, Methodology, Resources, Supervision, Validation, Visualization, Writing – review & editing. SB: Conceptualization, Funding acquisition, Methodology, Resources, Supervision, Visualization, Writing – review & editing.
